# Supplementary material for: Development of chemokine network inhibitors using combinatorial saturation mutagenesis
Source: Commun Biol. 2025 Apr 3;8:549. doi: 10.1038/s42003-025-07778-6 (PMC11969024; doi:10.1038/s42003-025-07778-6)
Supplement: Supplementary file 4 — Supplementary Data 2 [file 42003_2025_7778_MOESM4_ESM.zip › SupplementaryData3/scripts/xAF3.tar!/./script/analysis/JK/JK_ExactPvalues.docx]

Supplementary Table: Exact P values for Arpeggio bond numbers, comparisons to
control

| Test | Control | Number of data points | P value | Bond type | Amino acid type |
| --- | --- | --- | --- | --- | --- |
| HD2CM304 | HD2 | 46 | 1.74e-03 | all | all |
| HD2CM307 | HD2 | 46 | 5.95e-03 | all | all |
| HD2CM304 | HD2 | 46 | 2.13e-02 | hydrophobic | R3 |
| HD2CM307 | HD2 | 46 | 8.59e-03 | hydrophobic | R3 |
| HD2CM304 | HD2 | 46 | 4.02e-04 | hydrophobic | R5 |
| HD2CM307 | HD2 | 46 | 8.90e-05 | hydrophobic | R5 |
| HD2CM304 | HD2 | 46 | 2.81e-02 | hydrophobic | R6 |
| HD2CM304 | HD2 | 46 | 3.96e-08 | hydrophobic | R12 |
| HD2CM307 | HD2 | 46 | 1.12e-02 | hydrophobic | R15 |
| HD2CM304 | HD2 | 46 | 1.22e-04 | hydrophobic | all |
| HD2CM307 | HD2 | 46 | 7.07e-03 | hydrophobic | all |
